# Analysis of Protein Inhibitors of Trypsin in Quinoa, Amaranth and Lupine Seeds. Selection and Deep Structure–Function Characterization of the *Amaranthus caudatus* Species

**DOI:** 10.3390/ijms26031150

**Published:** 2025-01-28

**Authors:** Martha Hernández de la Torre, Giovanni Covaleda-Cortés, Laura Montesinos, Daniela Covaleda, Juan C. Ortiz, Jaume Piñol, José M. Bautista, J. Patricio Castillo, David Reverter, Francesc Xavier Avilés

**Affiliations:** 1 Facultad de Ciencias Forestales, Universidad de Concepción, Concepción 4030000, Chile; marhernandez@udec.cl; 2 Department of Chemical, Biological and Environmental Engineering, Universitat Autònoma de Barcelona, 08193 Barcelona, Spain; gcovaledacortes@gmail.com; 3 Institute of Food and Agricultural Technology-CIDSAV-XaRTA, University of Girona, 17004 Girona, Spain; laura.montesinos@udg.edu; 4 Institut de Biotecnologia i Biomedicina, Departament de Bioquímica i Biologia Molecular, Universitat Autònoma de Barcelona, 08193 Barcelona, Spain; daniela.covaleda@uab.cat (D.C.); jaume.pinyol@uab.cat (J.P.); david.reverter@uab.cat (D.R.); 5 Institut de Biotecnologia i Biomedicina, Departament de Genètica i Microbiologia, Universitat Autònoma de Barcelona, 08193 Barcelona, Spain; juancamilo.ortiz@uab.cat; 6 Department of Biochemistry and Molecular Biology, Universidad Complutense de Madrid, 28040 Madrid, Spain; jmbau@ucm.es; 7 Departamento de Ciencias Nucleares, Escuela Politécnica Nacional, Quito 170143, Ecuador; pesd2011@gmail.com

**Keywords:** plant trypsin inhibitors, quinoa, amaranth, lupine seeds, *Amaranthus hybridus*, *Amaranthus caudatus*, HPLC, MS and X-ray analysis, structure–function characterisation, plant defence

## Abstract

Protease inhibitors are biomolecules with growing biotechnological and biomedical relevance, including those derived from plants. This study investigated strong trypsin inhibitors in quinoa, amaranth, and lupine seeds, plant grains traditionally used in Andean South America. Amaranth seeds displayed the highest trypsin inhibitory activity, despite having the lowest content of aqueous soluble and thermostable protein material. This activity, directly identified by enzymatic assay, HPLC, intensity-fading mass spectrometry (IF-MS), and MS/MS, was attributed to a single protein of 7889.1 Da, identified as identical in *Amaranthus caudatus* and *A. hybridus*, with a *K_i_* of 1.2 nM for the canonical bovine trypsin. This form of the inhibitor, which is highly homogeneous and scalable, was selected, purified, and structurally–functionally characterized due to the high nutritional quality of amaranth seeds as well as its promising agriculture–biotech–biomed applicability. The protein was crystallized in complex with bovine trypsin, and its 3D crystal structure resolved at 2.85 Å, revealing a substrate-like transition state interaction. This verified its classification within the potato I inhibitor family. It also evidenced that the single disulfide bond of the inhibitor constrains its binding loop, which is a key feature. Cell culture assays showed that the inhibitor did not affect the growth of distinct plant microbial pathogen models, including diverse bacteria, fungi, and parasite models, such as *Mycoplasma genitalium* and *Plasmodium falciparum*. These findings disfavour the notion that the inhibitor plays an antimicrobial role, favouring its potential as an agricultural insect deterrent and prompting a redirection of its functional research.

## 1. Introduction

Protein protease or peptidase inhibitors (PPIs) are essential bioactive molecules throughout living organisms. These inhibitors regulate key biological processes such as embryogenesis, aging, pathologies, parasitism, and infections, making them biologically and biotechnologically significant [1]. In plants, PPIs play critical roles in seed germination, growth, flowering, maturation, senescence, and defence against predators, pests, and environmental stressors [2,3,4,5]. Their controversial anti-nutritional properties and health impacts also warrant attention [6]. Understanding the structure and function of PPIs is vital for advancing agriculture, medicine, and biotechnological applications.

Despite considerable knowledge on plant PPIs, further research is needed, particularly given their importance in agriculture, nutrition, and health. The MEROPS database classifies PPIs into 35 families across 108 species [7]. Commonly studied crops like *Solanum tuberosum* (potato), *Glycine max* (soybean), and *Triticum aestivum* (wheat) each contain over 250–300 homologs of known and putative PPIs, indicating their genomic significance. PPIs are grouped by specificity, with serine protease inhibitors being the major group, while other categories, such as cysteine-, aspartic-, or metallo-protease counterparts, have a more limited representation in plants. Structurally, MEROPS identifies 40 folding types for plant PPIs, while a simplified classification highlights 12 major fold types, including phytostatins, serpins, Kunitz type, potato type 1, Bowman Birk type, and cyclotides [2,7]. Although significant progress has been made, more work is needed to fully integrate structural knowledge for biotechnological and therapeutic advances.

Among traditional Andean crops, *Chenopodium* spp. (quinoa), *Amaranthus* spp. (amaranth), and *Lupinus mutabilis* (Andean lupin) are nutritionally valued for their high protein content and balanced amino acid profiles [8,9]. These crops also contain significant levels of serine protease inhibitors [9]. Because of their potential utility, controversial value, and molecular differentiation between variants, we are studying in the seeds of such species the content and properties of protein inhibitors of proteases, and particularly of serine proteases, found there at significant levels and potency [9]. In this study, we analysed seeds from two amaranth species, *A. hybridus* (also known as sangorache) and *A. caudatus* (known as kiwicha), as well as from quinoa (*Chenopodium quinoa* L.) and lupine (*L. mutabilis*) for their trypsin-inhibitory activity. Both amaranth seeds demonstrated the highest inhibitory potency, with greater molecular homogeneity and scalability compared to quinoa and lupine.

Using an affinity proteomics intensity-fading MS approach [10], we identified active inhibitors in all extracts and isolated the amaranth inhibitors. These inhibitors, identical in mass and fingerprints, were further purified. The inhibitor from *A. caudatus*, termed ATSI [11], was complexed with bovine trypsin and structurally characterized via X-ray crystallography, revealing its inhibitory mechanism. We also investigated the ability of such inhibitor(s) to affect the growth of microbial species responsible for plant diseases, as described for other protease inhibitors [5]. This work aimed to enhance understanding of plant PPIs, paving the way for future studies and applications in nutrition and biotechnology/biomedicine.

## 2. Results

### 2.1. Trypsin Inhibition Dose–Response Curves, Inhibitory Capabilities, and Identification of Inhibitory Species

Relying on previous prospective analysis, aqueous extracts from the seeds of the four selected distinct Latino American plants, *Chenopodium quinoa* (quinoa), *Amaranthus hybridus* (sangorache), *Amaranthus caudatus* (kiwicha), and *Lupinus mutabilis* (chocho, Andean lupine), were analysed to determine the content of peptide/protein inhibitors of trypsin, as this is one of the main and more representative serine proteases. Firstly, the different seeds, after being milled into fine flours, were defatted with 1-propanol (1:4 *v*/*v*, solid/liquid), stored at 4 °C in dry state, and extracted before analysis with a TrisHCl-NaCl buffer (pH 8.0). Subsequently, the protein content of the extracts was analysed by the bicinchoninic acid chemical method. The extracts displayed considerable protein contents, particularly in the case of the lupine seeds, as shown in Table S1. These high contents were essentially maintained after the application of two alternative clarification treatments, either with trichloroacetic acid at 2.5% (*w*/*v*) or heating at 65 °C, both of which have been traditionally used to remove unwanted materials.

**Figure 1:**
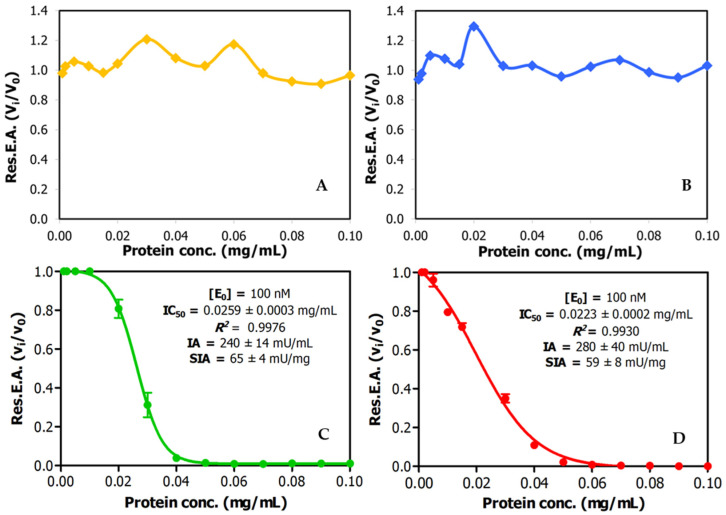
Dose–response relationships for trypsin inhibitory activity in crude plant seed extracts and derivation of IC_50_ values. Effects of different doses of crude extracts on trypsin activity: (**A**) quinoa; (**B**) lupine; (**C**) *Amaranthus hybridus* (sangorache); and (**D**) *Amaranthus caudatus* (kiwicha). The substate was 1.0 mM of bApNA. Res.E.A.: residual enzymatic activity. IA: inhibitory activity. SIA: specific inhibitory activity.

However, when titration activity curves of trypsin inhibitory capability were obtained by adding increasing volumes of these extracts on model bovine trypsin, this biological activity was only initially detected in the derived plots for the two tested amaranth species, *A. hybridus* and the *A. caudatus*, but not for quinoa or lupine, as shown in Figure 1. This indicates that the inhibitory capability of the studied inhibitory species and their impact on related protein content is significant in the two amaranth species but less so in the two other ones. Interestingly, from these dose–response or titration curves, IC_50_ parameters were derived for the *A. hybridus* and *A. caudatus* samples, with values of 0.0259 +/− 0.0003 mg/L and 0.023 +/− 0.0002 mg/mL. This is indicative of very high inhibitory capabilities for both extracts and the inhibitory molecules that they contain, as well as predicting their low *K_i_* values, as confirmed in subsequent experiments.

**Figure 2:**
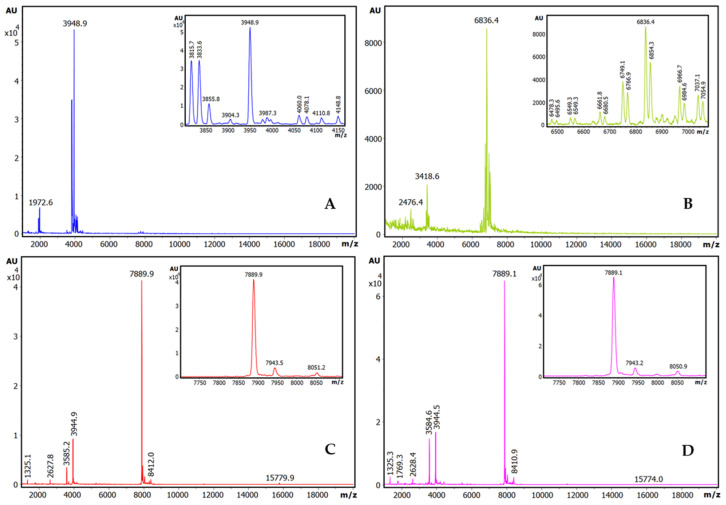
Identification of trypsin inhibitors by intensity-fading MALDI-TOF MS in crude plant seed extracts on bovine trypsin-glyoxal Sepharose^R^. Stack view of the mass spectra from plant extracts corresponding to the elution fractions of IF-MALDI-TOF MS analyses on bovine trypsin-glyoxal Sepharose^®^ CL4B. (**A**) Quinoa. (**B**) Lupine. (**C**) *A. hybridus* (sangorache). (**D**) *A. caudatus* (kiwicha). The elution from the affinity matrix was performed with 0.5% *v*/*v* TFA. Assays were carried out in duplicate, at room temperature. See details in the Section 4.

Additionally, the extracts were analysed using our more sensitive and previously reported affinity-based intensity-fading MALDI-TOF.MS approach (IF-MS) [10,12,13], combining the concentration action of affinity capture in spin microcolumns or microbeads of glyoxal Sepharose CL4B matrix derivatised with trypsin [10,13] with high-sensitivity analysis by HPLC and mass spectrometry, overall unveiling a more positive view of all the extracts. Thus, such binding analyses clearly indicated the occurrence of distinct strong trypsin-binding molecules in all such seeds, with m/z values ranging from 3900 to 7900, as shown in Figure 2. Our efficient affinity matrix derivatization procedure allowed us to achieve a high content and activity for the immobilised trypsin (as shown in Table S2), which surely contributed to the clear visualization of serine protease inhibitors from plant species.

To better identify the molecules responsible for such strong binding capabilities (probably inhibitors), the same affinity-based IF-MS approach, was applied at semi-preparative levels and in larger centrifugal microcolumns to the seed extracts of the four plant species, using the trypsin-Sepharose CL4B matrix as the capturing agent. As seen in Figure 2, the main components visible after IF-MS appear at 3948.9, 6836.4, 7889.9, and 7889.1 mz for each species, although some spectral heterogeneity appear for the quinoa and lupine species, which are probably real but are also indicative of the low concentration of the captured molecules that are present in the seeds. The lower-intensity spectral signals at about 2× and 0.5× the main *m*/*z* values are clearly visible for *A. hybridus* and *A. caudatus*, signals that most probably corresponding to double- and half-charged MALDI signals, which are also present in the other two plants (particularly for the 2× values). To be confident of the genuine origin of the visualized main mass spectrometry signals, the MS-MALDI spectra of the crude extracts of *A. caudatus*, of the different washes, and of the final eluted sample (in the present case, using acid-based detachment of the inhibitors from the matrix) were compared, as displayed in Figures S1 and S2 It can be seen that none of the signals that appear in the final eluted sample, besides the main one at 7889.1 *m*/*z*, are visible, neither at the initial crude samples nor at any of the washes: i.e., this occurred for the 8840 *m*/*z* signal, which is visible in both the crude sample and in the non-binding eluate. By contrast, due to equivalent reasons, the 8337 *m*/*z* signal, although of minor occurrence and only present in some batches, it could be a genuine binder. All of these analyses stimulated us to concentrate research on the inhibitor from the *A. caudatus* species, one of the amaranths that garners more agricultural and nutritional interest nowadays.

### 2.2. Purification and Mass Characterization of Natural ATSI

**Figure 3:**
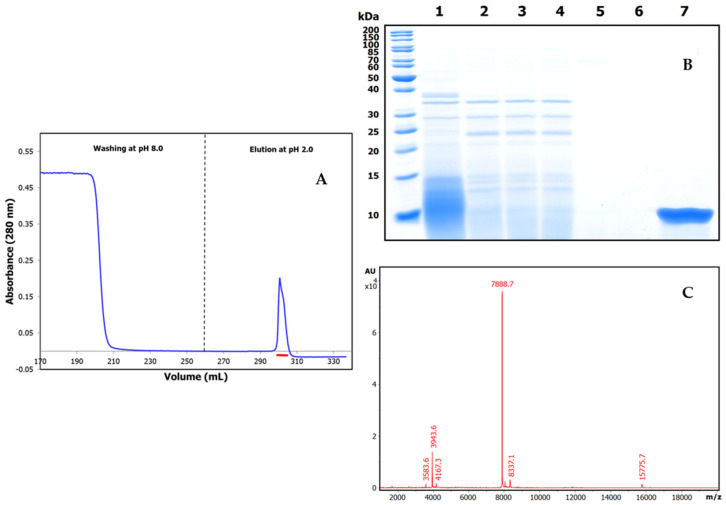
Purification and basic characterization of natural *A. caudatus* trypsin inhibitor (ATSI). (**A**) Affinity chromatography of the crude extract of *A. caudatus* on a bovine trypsin-glyoxal Sepharose^®^CL4B column. Equilibration, loading, and washing with a buffer at pH 8.0 and at room temperature is shown on the left. The elution buffer at pH 2.0 released the ATSI peak of trypsin inhibitory activity, as is shown on the right. (**B**) SDS-PAGE analysis of the trypsin affinity chromatography fractions of ATSI, run in 15% acrylamide gel. Lanes 1 and 2: loading and get-through fractions; lanes 3 to 6: washing fractions at n° 1, 4, 8, and 12, respectively; lane 7: inhibitory ATSI elution peak. (**C**) MALDI-TOF.MS spectrum of purified ATSI. Spectral acquisition, using DHAP as a matrix.

Once the *Amaranthus caudatus* trypsin inhibitor of 7889.1 *m*/*z*, abbreviated here as ATSI, had been visualized as the most promising target among those assayed, we purified it with enough (on a large scale) quantities from trustable and certificated seeds. For this, we extended the analytical affinity-based approach to the preparative level, using a 25 mL trypsin-glyoxal Sepharose CL4B column with a high load of extract. We applied a slightly alkaline buffer (pH 8.0) to the column and washed it, followed by elution in acidic conditions (pH 2.0)The chromatographic profile, shown in Figure 3A, indicated the clean separation of a fraction with high trypsin inhibitory capability, confirmed to be practically pure by RP-HPLC (C8), PAGE analysis, and high resolution MALDI-TOF.MS spectrum, shown in Figure 3B, C. In these conditions, the spectrum confirmed a molecular mass of 7888.7 *m*/*z* for its single-charged species, with signals at 3943.6 and 15,775.7 for the 2.0× and 0.5× charged ones.

### 2.3. Determination of Inhibitory Activities, K_i_ Value, Number of Cysteines, and Top-Down MS Sequencing Analysis

**Figure 4:**
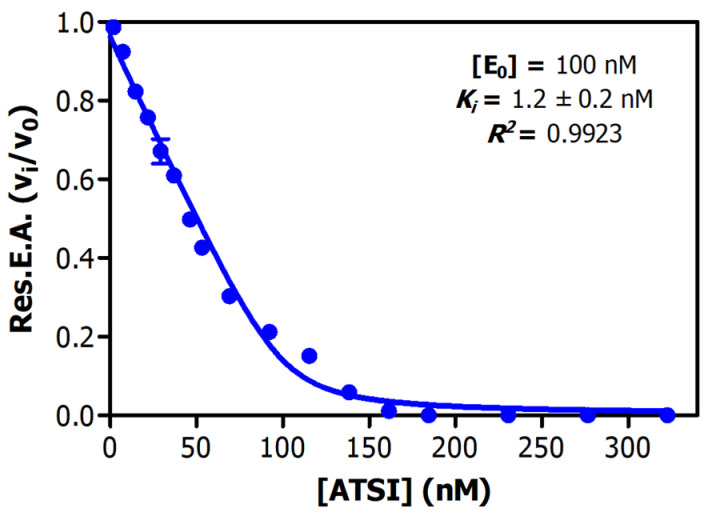
Equilibrium dissociation constant (*K_i_*) of ATSI against bovine trypsin. Assays performed with 1.0 mM bApNA substrate, in 20 mM Tris-HCl buffer pH = 8.0 and 150 mM NaCl 20 mM CaCl_2_, with 0.05% *v*/*v* Triton X-100 as activity buffer, at 37 °C. Pre-incubation: 15 min at 37 °C. See experimental details and data treatment in the Section 4.

Analysis of the inhibitory activities along the purification procedure evidenced that ATSI was purified about 75-fold from the crude extract, achieving a specific inhibitory activity of 4.5 +/− 0.3 U/mg on bovine trypsin (see Table 1). Deduction of the enzymatic parameters of ATSI against this enzyme at different protein inhibitor/enzyme ratios was achieved following the Morrison approach [14,15], and after enzyme titration, this gave rise to an equilibrium dissociation constant value, *K_i_*, of 1.2 +/− 0.2 nM, at 37 °C and pH 8.0 (Figure 4). It is noteworthy that the derived graphical plot was typical of tight-binding inhibitors when representing vi/vo against inhibitor concentration (at nanomolar ranges). Interestingly, the purified protein displayed strong inhibitory capability against α-chymotrypsin and subtilisin A, features also reported for ATSI [11].

**Figure 5:**
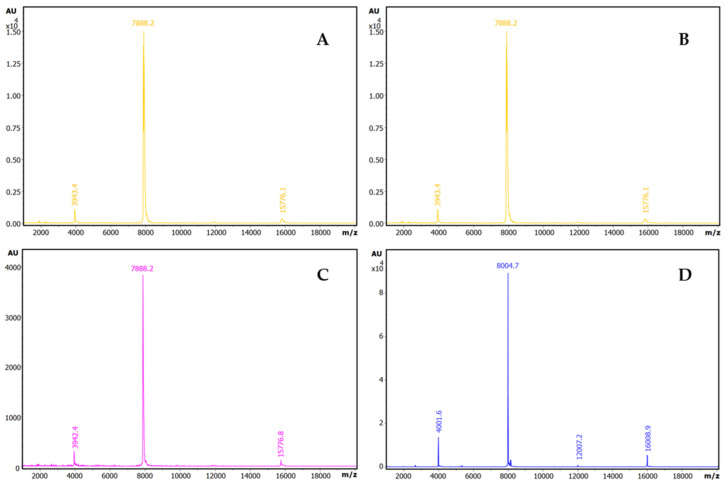
Determination of total free and paired cysteine residues of ATSI. MALDI-TOF MS spectrum (**A**), (**B**) of native ATSI; (**C**) after S-carbamidomethylation; (**D**) after reduction and S-carbamidomethylation. DHAP was used as a matrix.

On the other hand, we analysed the number of cysteine residues in this ATSI molecule by reduction or by reduction and derivatisation with iodoacetamide and MALDI-TOF.MS comparative analysis of the natural and modified variants of the molecule, as shown in Figure 5. From this, it appeared that ATSI became double carbamidomethylated with the alkylation agent, indicative of the occurrence of two cysteines in the natural molecule as a disulfide-linked cystine, with no free cysteine. The subsequent evidence on the occurrence of such two cysteines in the molecule, at positions 4 and 49, obtained via the resolution of its three-dimensional structure, confirmed that they are disulfide bridged.

**Figure 6:**
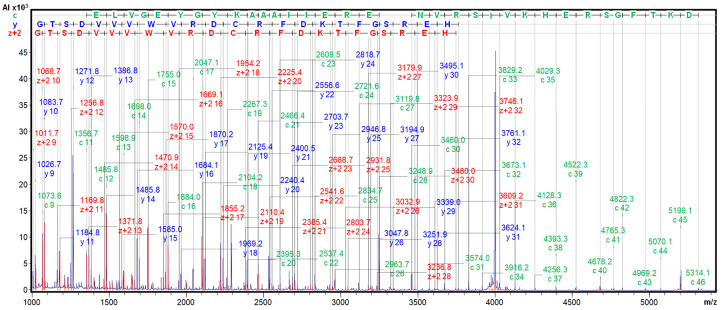
ISD MALDI-TOF MS analysis of ATSI. The ISD fragmentation ladder spectrum of purified ATSI (after HPLC-C8 polishing) was performed on the reduced and S-carbamidomethylated ATSI using 2,5-DHB as an MALDI matrix. See details in the Section 4.

We confirmed the identity of our purified protein with the *A. caudatus* inhibitor identified by J Hejgaard et al. (1994) [11] by MALDI-TOF.MS/MS fragmentation, with ISD and CID, in the presence of the 2,5-DHB matrix. The derived spectra clearly displayed long fragmentation ladders, with c, y, and Z+2 fragments from positions 9–46, 9–31, and 9–32, as shown in Figure 6 and Figure S3. Very similar spectra were generated from the *A. hybridus* inhibitor. All of this, together with precise identical molecular masses of 7888.7 *m*/*z*, allowed us to confirm the identity of the here-isolated ATSI sequence with the one initially reported above, as well as that of the sequence between the ATSI inhibitors found in the two amaranth species. Inhibitors purified from both amaranth species were identical. All our data, along with the precise identical molecular mass of 7888.7 *m*/*z*, support the identity of the here-isolated ATSI sequence protein being the same as the previously reported inhibitor [11].

### 2.4. Formation and Isolation of the ATSI-Bovine Trypsin Complex

**Figure 7:**
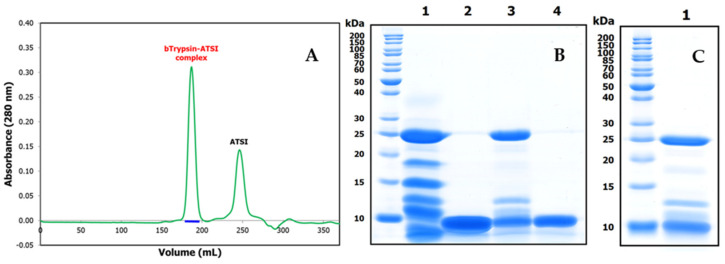
Size exclusion chromatography of the ATSI-bTrypsin complex on a HiLoad Superdex75 column, and SDS-PAGE analysis of complex purification. (**A**) Separation of the (first) peak of the complex from the (second) peak of excess ATSI was achieved in 20 mM Tris-HCl, 150 mM NaCl, and 20 mM CaCl_2_, at pH 8.0 and room temperature. (**B**) SDS-PAGE analysis at 15% acrylamide. Lanes 1 and 2: bovine trypsin and ATSI after 30 min incubation (alone) in activity buffer. Lane 3: bovine trypsin–ATSI complex after 30 min incubation in a 1:2 molar mixture of them in activity buffer and purification through Superdex75. Lane 4: ATSI in excess recovered by Superdex75. (**C**) Analysis of the complex from crystals before X-ray diffraction analysis.

To start analyzing in detail the complexes that ATSI could establish with standard serine proteases, we assayed the formation and isolation of a binary complex of the inhibitor with bovine trypsin as an experimental model. This was achieved by mixing both molecules in the presence a 2:1 molar excess of ATSI over trypsin, at pH 8.0 and 25 °C, in 20 mM Tris-HCl, 150 mM NaCl, and 20 mM CaCl_2_. After incubating the complex for 30 min., subsequent gel filtration chromatography of the mixed sample through a Superdex 75 High load column, in the same buffer, allowed a clean separation of the complex from the excess inhibitor and the purification of the former, as shown in the SDS-PAGE (see Figure 7) and RP-HPLC results. It is noteworthy that, in spite of elution occurring in a very symmetric gel filtration peak, the sample from the complex displayed in the electrophoresis two additional bands of intermediate masses between the ones from trypsin and ATSI that resembled the formation of autolytic large and limited trypsin fragments, previously well reported for trypsin [16,17]. This could indicate that the overall Stokes radius of the complex is not affected by such cleavages, if by any, and that the conformation and main structure–function determinants of trypsin, when held by disulfides, are not grossly affected. With the prepared complex readily crystallised, this allowed for the clean resolution of its three-dimensional structure (see below). It is noteworthy that the potentially remaining trypsin activity of the complex was found to be null, and the X-ray electron density maps did not reflect any evidence of cuts/discontinuities at the protein inhibitor main chain (see next section). This probably reflects the fact that trypsin (isolated or in complex) mainly autolysed when mixed with the SDS buffer of PAGE, but it was intact within the analysed complex.

### 2.5. Three-Dimensional Crystal Structure Analysis of the ATSI-bTrypsin Complex

In order to facilitate crystallization, the purified ATSI-bTrypsin complex was concentrated to 16.0 mg/mL by centrifugal ultrafiltration, and at that time, the purification buffer was exchanged with 5 mM Tris-HCl and 50 mM NaCl (pH 8.0). The freshly prepared and concentrated complex was subjected to crystallization assays, giving rise to well-formed protein crystals. The three-dimensional structure of the crystallized complex, after analysis with an Alba Synchrotron X-ray beam, was obtained at 2.85 Å resolution. The quality of the electron density map and excellent crystallographic parameters (Table S3), allowed for the perfect trace of side chains along the whole proteins, particularly regarding the enzyme subsites and the inhibitory “reactive” site of ATSI.

**Figure 8:**
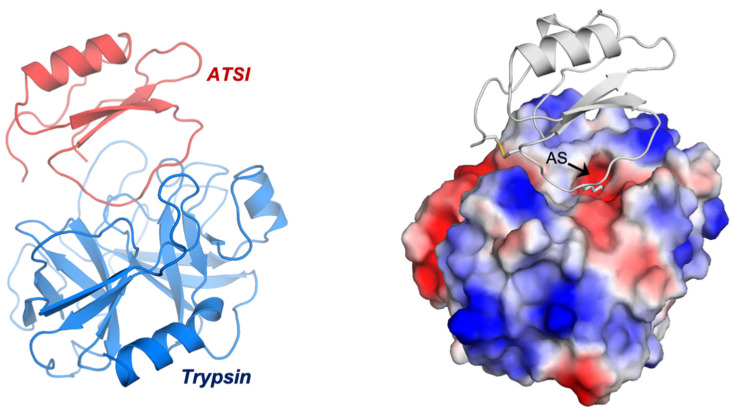
Three-dimensional structure of ATSI in complex with bovine trypsin. Depiction of the complex structure shown in ribbons (**left**), as well as surface-charged representations (**right**). The latter depicts the penetration of the long binding/reactive loop of the inhibitor within the active site of the enzyme, including “reactive” Lys 45, and the presence of a disulfide bridge between Cys4 and Cys49 in ATSI. The black arrow indicates the trypsin active site entrance.

The asymmetric unit of the crystal contained eight complexes of ATSI-trypsin, basically all sharing a similar three-dimensional structure (Figure 8). The structure of bovine trypsin in complex with ATSI aligns well with other trypsin structures deposited in the PDB database. Despite the small size of ATSI, its crystal structure contains some secondary structural elements, namely a two stranded parallel ß-sheets and a small α-helix, which are able to form a hydrophobic core and stabilize its fold. The active part of the molecules corresponds to the loop connecting the two ß-strands, which is called “binding loop”, because it contains all the elements necessary to interact and inhibit serine proteases such as chymotrypsin, subtilisin, or, as in this instance, trypsin. ATSI from amaranth also contains one disulfide bridge (Cys4–Cys49), which helps to stabilize the overall structure and, in particular, to maintain the conformation of the binding loop in the right orientation for inhibition.

**Figure 9:**
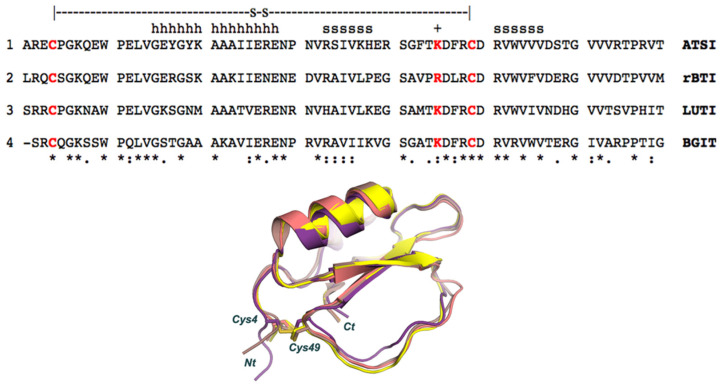
Aligned sequences and overlapped conformations of ATSI with the homologous inhibitors of serine proteases with known three-dimensional structures (about 41.5% minimal sequence identity). (1) sp|P80211|, ATSI from *Amaranthus caudatus*; (2) tr|Q9S9F3|, rBTI from Buckwheat; (3) sp|P82381|, LUTI from *Linum usitatissimum* (Flax) (var. *Linum humile*); and (4) tr|Q7M1Q1|, BGIT from *Momordica charantia* (Bitter gourd) (Balsam pear). h refers to residues in α-helix; s refers to residues in ß-strand. The + sign refers to the reactive/active site (cleavable site) of the inhibitor. The disulfide bridge connection is shown using a stick representation.

A structural Blast analysis applied to the whole PDB database indicated that the three closest structural protein homologues of amaranth ATSI in the data bank are: (1) rBTI from buckwheat (PDB code 3RDZ, with a rmsd of 0.69 Å for 67 aligned residues); (2) LUTI, the *Linum usitatissimum* trypsin inhibitor (PDB code 1dwm, with an rmsd of 1.06 Å for 67 aligned residues); and (3) BGIT, a trypsin inhibitor from bitter gourd (PDB code 1vbw, with a rmsd of 0.66 Å for 66 aligned residues) (Figure 9).

The low rmsd value in these instances evidence that such proteins share a similar structural fold and are members of the potato inhibitor I family of serine protease inhibitors. As shown in Figure 9, ATSI nicely aligns with all such trypsin inhibitors at the level of primary, secondary, and tertiary structures. It is also noteworthy that there is a close conformational overlap of the long inhibitory/reactive site loop of ATSI and those of the other three inhibitors of very similar size (around eight residues) and conformation, which protrudes towards the active site of the receptor enzyme, as displayed in Figure 8 and Figure 9.

### 2.6. Structure–Function Details of the ATSI-Trypsin Complex

**Figure 10:**
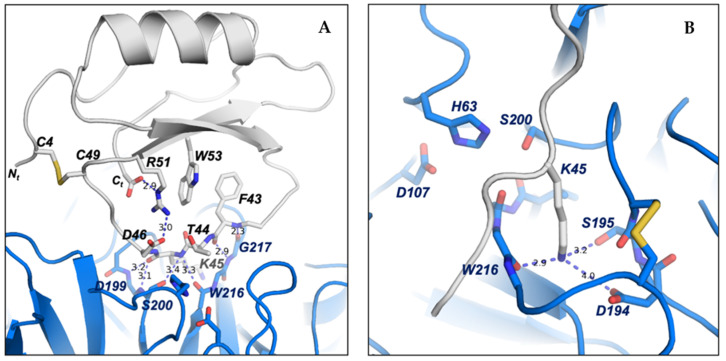
Detailed view of the fundamental three-dimensional structural elements of ATSI-trypsin complex. (**A**) Detailed representation of the contacts in ATSI used to shape the “reactive” loop into a correct orientation for binding to trypsin. Side chains are labelled and shown using a stick representation. The residues essential for the mutual recognition of the inhibitor and enzyme and for the establishment of the transition state are shown. (**B**) Binding of the “reactive” Lys45 of ATSI (in grey) inside the trypsin-specific pocket. Numbered dotted lines indicate important polar interactions and bond distances.

To maintain the correct conformation of the binding loop of ATSI for the substrate-like inhibition of trypsin, several contacts are formed inside ATSI between the residues of the ß2-strand and the binding loop. It is noticeable that Arg51 plays a role in ATSI, sandwiched as it is between a hydrophobic interaction with Trp53 and an electrostatic interaction with the C-terminal carboxylate end of ATSI (2.9 Å). Arg51 also forms a salt bridge with Asp46 from the binding loop (3.0 Å), which, in addition to forming a hydrophobic interaction with Phe43, helps to maintain the correct conformation of the binding loop for the right orientation of the substrate-like Lys45 to fit into the binding pocket of trypsin (Figure 10A). All these interactions described in ATSI from amaranth are conserved in other homologues of the family, such as lute or rBTI [18], whose complex three-dimensional structures are known. In other members of the family, some substitutions in this region can be observed, but in all cases, the fixed conformation of the binding loop, which is essential for the inhibition, is maintained.

The low binding inhibitory constant of ATSI for bovine trypsin, indicative of tight binding, can be basically attributed to two major factors: first, to the perfect fitting of the substrate-like Lys45, which potentially constitutes the “reactive” cleavage site, into the negatively charged binding pocket of bovine trypsin; and second, to the hydrogen bond network formed between the main chain residues of the ATSI binding loop and the active site surface of trypsin. The negatively charged binding pocket of bovine trypsin is formed by the side chains of Asp194 and Ser195 and by the carboxylate oxygen of Trp216. Essentially, the substrate-like side chain of the ATSI Lys45 is buried in the trypsin substrate pocket with its positively charged ε-amino group positioned at hydrogen bond distance to the carboxylate oxygen of Trp216 (2.9 Å), to the side chain of Ser195 (3.2 Å), and to the negatively charged Asp194 (4.0 Å), conducting a strong buried electrostatic interaction (Figure 10B).

However, the reason for the non-productive enzymatic binding of ATSI, that makes difficult its cleavage by bTrypsin, as well as external cleavages promoted by this enzyme, can be definitively attributed to the extensive network of hydrogen bonds between the ATSI binding loop and the active site region of trypsin, which traps ATSI in a substrate-like transition state of the reaction (Figure 10A). In particular, these interactions are conducted by: the carboxylate oxygen and amino main chain of Phe43 with the respective amino and carboxylate oxygen of Gly217 (2.4 Å and 2.9 Å, respectively); the amino main chain of Lys45 with the amino of Ser200 (3.4 Å) and carboxylate oxygen of Ser215 (3.3 Å); the carboxylate oxygen of Lys45 with amino groups of Gly199 (3.2 Å) and Ser200 (3.1 Å); and the amino group of Phe47 with the carboxylate oxygen of Phe47 (3.1 Å) of trypsin. Also, at the edges of the ATSI binding loop, other hydrogen bonds are conducted between main-chain Glu39, Arg40, and Cys49 and the side chains of Ser218 (2.9 Å), Lys225 (2.8 Å), and Tyr45 (3.4 Å) of trypsin, respectively. All these interactions help to shape and maintain the correct conformation of the binding loop and are observed in most of the complexes of the asymmetric unit of the crystal. This extensive network of hydrogen bond interactions inhibits the catalytic reaction by trapping ATSI as a non-productive substrate-like inhibitor.

### 2.7. Assaying the Capabilities of ATSI to Act as an Antimicrobial in Cell Cultures

To further assess the functional properties of ATSI, we analysed those that could be related to the defence of the plant against microbial attacks, as has often been suggested generically as well as specifically for plant protease inhibitors [3,5,19,20], and to potential biotech/biomed applications. Thus, the growth of a series of bacterial cell cultures from well-known plant pathogens, like *Erwinia amylovora* (Ea), *Xanthomonas arboricola* PV. pruni (Xap), and *Pseudomonas syringae* pv. Tomato (Pto), were analysed at different concentrations of ATSI, from 1 to 100 µM, regarding the final concentrations of the inhibitor, following the drop plate assay on agar approach [21], at 28 °C and pH 7.4, as shown in Figure S4 for the highest concentration assays. In parallel, as a reference, the same assays were made with the antibacterial peptides BP100 and BP178, two well-known synthetic antimicrobial reference peptides for these species [22]. No inhibitory effect on the growth of such bacterial cultures was observed among the tested ATSI concentrations, whilst in the tested conditions, the reference peptides strongly affected the bacterial growth.

The assays were also expanded to three plant pathogenic fungi known to be important vegetal pests, *Fusarium oxysporum*, *Penicillium expansum*, and *Botrytis cinerea*, in similar concentrations to ATSI, at 23 °C and pH 7.4, and using a distinct reference antifungal peptide BP15 [23], within a similar range of concentrations. Again, no inhibitory effects of the ATSI on the growth of such fungi were observed here, whilst positive effects were evident for the antifungical reference compound, as shown in Figure S5. Also, to confirm this, the assay was repeated in the presence of 200 µM ATSI against *Fusarium oxysporum*, taking care this time to avoid applying freezing/defrosting steps to the stored samples that could damage the ATSI, but again, we received a negative result.

Also, assays were made using *Mycoplasma genitalium* as a model microorganism, one which is evolutively and functionally unrelated to the previous microbial models. Mycoplasmas are minimal cells with very limited biosynthetic pathways, and the presence of very promiscuous membrane transporters makes them quite permeable to external polar and non-polar molecules [24,25]. In addition, *M. genitalium* is an emerging human pathogen, and there has been increasing interest in developing non-antibiotic therapies against this microorganism. When ATSI was included in *M. genitalium* cultures in the range of 5–100 µM, a small increase in the duplication time of this microorganism was noticeable, and cells reached the stationary phase of growth 15 h later than cells without ATSI (Figure S6A). However, cell densities in the stationary phase were nearly identical both in the presence and in the absence of ATSI, concluding that this small polypeptide does not significantly inhibit the growth of mycoplasmas (Figure S6B). In contrast, when *M. genitalium* cells were incubated with a small amount of a protein extract from the marine invertebrate *Nerita peloronta*, which is very rich in protease inhibitors [10], a strong inhibitory activity on the mycoplasma growth was observed (Figure S6A,B).

Additionally, and to achieve a more complete view, antimicrobial assays were conducted with ATSI on the microbial parasite *Plasmodium falciparum*, a protozoan of high biomedical impact, to expand our view of the properties and potentialities of the inhibitor. Initial results were negative, with a non-significative reduction in the growth of this microbe observed in the presence of a wide range of assayed concentrations of this trypsin inhibitor, from 0 to 222 µM (Table S4 and Figure S7), using chloroquine as a reference anti-*Plasmodium* compound. It is noteworthy that at the higher assayed concentration of 444 µM, haemolysis of the erythrocytes included in the cell culture took place, indicating a potentially damaging action of ATSI in mammalian cells that should be taken into account.

## 3. Discussion

The interest in developments regarding proteolytic enzymes (proteases) and their inhibitors remains strong due to their diverse biological functions and their potential in biology, biotechnology, and biomedicine. This also applies to plant proteases and their inhibitors, both for their intrinsic properties and their proven or potential roles in biotechnological applications, such as plant protection against parasites and predators [3,4,5,26], and in human biomedical issues and applications, as in digestive dysfunctions [6], cancer development [27,28], and anti-microbial strategies [5,29], among others. The great potency of such molecules makes it advisable to carefully evaluate their properties, generically and as bioactive compounds, and the limits of their use and applications. This is particularly valid for cereals, pseudocereals, and Leguminosae and their seeds, which are usually rich in protease inhibitors, mostly of proteinaceous nature.

Additionally, at the nutritional level, such developments regarding several seeds of these plants overlaps with interest in the advised and increasing use of them in the human diet because of their high and equilibrated protein content and essential constituent amino acids; also, they contain other beneficial constituents and lack gluten in pseudocereals and Leguminosae [30]. Therefore, the consumption and cultivation of these plants is steadily increasing nowadays. However, given that warnings have been issued about the potential anti-nutritional properties of the abundant protease inhibitors found in plant seeds [6,31], an important question involves to what extent such concern has to be extended to them.

Quite a number of publications, mainly since the 1990s, reporting the occurrence of protease inhibitors in the pseudocereals *Chenopodium quinoa* (quinoa) and *Amaranthus* sp. (amaranth), as well as in Leguminosae, have appeared in the literature [32,33,34,35]. This has been confirmed in our study through the analysis of seed extracts from three varieties commonly used in the Andean region, using our affinity/IF-MS proteomics approach. Thus, in such extracts, several small proteins with masses of around 3949 Da. for *Chenopodium quinoa* (quinoa) and 6838 Da for *Lupinus mutabilis* (lupine) were found, as well as a single form of about 7890 Da. for *Amaranthus hybridus* (sangorache). Also, the extract from the seeds of this amaranth showed the highest trypsin inhibitory capability among the three species, although the highest protein content was clearly assignable to the lupine samples. Such remarkable protein content has already been reported in lupine seeds, in which the presence of bitter alkaloids and antinutritional factors, as protease inhibitors, limits its consumption, requiring previous debittering and fermentation treatments [36].

Given the clear signals and MALDI-MS homogeneity of the molecular species that have appeared as responsible for the high inhibitory content (per gram of seeds) of *A. hybridus* (sangorache), we decided to extend the analysis to *A. caudatus* (kiwicha), a parent species of the same genus, because although both are of Andean origin, the first one is more commonly used at the level of consumption as leaves or for ornamental uses, whilst for the latter one, consumption as seeds and flour is more important. In America, *A. hypochondriacus* originates and is mainly used in Mexico, *A. cruentus* in Guatemala and southeast Mexico, and *A. caudatus* and *A. hybridus* in Andean South America [8,37,38]. The use of these and other related varieties is also common and rising in other parts of the world [8,37]. Remarkably, our analysis of the seed extracts again indicated a high (equivalent) trypsin inhibitory content for *A. caudatus*, as well as exactly the same molecular mass, molecular homogeneity, and protein sequence of its inhibitor by affinity, IF-MS, and MS/MS, as in the previously analysed *A. hybridus* species. It is noteworthy that our aim was to use certified seeds from a single country, produced in fairly similar climatic and cultivar conditions, to facilitate comparisons, and this method was followed for the four distinct seeds here investigated.

On light of the above results, we focused our attention on two previous publications in the field, one by Valdes-Rodriguez et al. (1993) [39] and one by Hejgaard et al. (1994) [11] on the initial characterization of trypsin inhibitors from amaranth seeds, the former from *A. hypochondriacus* and the latter from *A. caudatus*. Interestingly, in the first paper, the seeds were defatted with chloroform/methanol (2:1) before aqueous extraction, whilst in the second case, they were defatted with ethyl acetate, as well as being processed with different aqueous extraction and purification procedures. In the present work, we used a “greener organic solvent” for defatting [40], i.e., propanol-1, which is common nowadays. These variables are mentioned here because they could give rise to unwanted variability in the extraction and final heterogeneity/appearance of distinct protein inhibitors, as well as of isoforms, if present, requiring full characterization. It is noteworthy that we used trypsin as the major model for serine protease targets in the study, because it is the most selected one in the field, but we must keep in mind that protein inhibitors usually have a rather wide range of specificities for target proteases of the same catalytic class (i.e., chymotrypsin, elastase, and/or subtilisin), although in certain cases, they can be more restricted regarding the natural target enzyme.

Interestingly and relatedly, in both previous works, they found that the major form of trypsin inhibitor was as a sequence of about 69 residues, one from *A. hypochondriacus* and the other one from *A. caudatus*. Subsequently, for the first form, differences were found regarding the other form in positions 34 (Asp/Ser) and 59 (Tyr/Ser), as well as double positions, a probable indication of isoforms, for the *A. hypochondriacus* inhibitor for residues 41 (Ser/Tyr) and 65 (Thr/Tyr) (see Figure S8). In this work, Valdez-Rodriguez et al. (1999) [41] cloned and sequenced the cDNA of the trypsin inhibitor of *A. hypochondriacus*, corrected the former sequence, and found that the region encoding the mature protein had the same 69-residue sequence as the one found by Hejgaard et al. (1994) [11] in *A. caudatus*. However, in this later work, Valdez-Rodriguez et al., focusing on the duality in position 41 (Ser/Tyr), concluded that the sequence differences between the two publications and species could be due to the possible occurrence of two distinct gene copies of the protein inhibitor in the amaranth genome, because of the observation of distinct fragments when this cDNA was cleaved by restriction endonucleases in a Southern blot analysis.

In this respect, our experimental HPLC, IF-MS, comparative molecular masses, and long fragmentation ladder MS/MS analyses of the trypsin inhibitor fraction that we isolated from *A. caudatus* indicated that a single/unique molecular form of the trypsin inhibitor, of 7889.1 Da. and with 69 residues, was present in our seeds and extracts. Also, that such a form was identical in mass, sequence, and homogeneity to the form studied by us from *A. hybridus* in this work, found as a single form and characterised at these levels for the first time. For clarity, let us remember that these two species originated and are mainly cultivated in Andean countries, whilst the *A. hypochondriacus* one is centred in Mexico and Central and North America. Overall, and in agreement with our results and the above-described previous works, it can be suggested that a single form of this trypsin inhibitor with 69 residues is present and identical in *A. hybridus* and *A. caudatus* at the protein level and is also present in *A. hypochondriacus* but with minor differences in sequence, with the probable occurrence of a second isoform in the latter. The presence of second isoforms in *A. hybridus* and *A. caudatus* cannot be discarded given that the visualisation of isoforms, particularly for minor ones, could be dependent on the distinct defatting, extraction, and purification procedures used in different works, or even on the differential expression of genes (if double) in distinct growing environmental conditions and cultivars. Here, we did not consider as a factor the possible distinct releasing yields of potential isoforms from the affinity chromatographic matrix because this release is very clean and efficient, with very little unspecific binding of ATSI, as shown in the comparison between Figures S1 and S2.

The observed high homogeneity of the trypsin inhibitor from *A. caudatus* seeds, with the expected easy scalability, and its strong inhibitory potency versus the enzyme, with *K_i_* at a low nanomolar level for trypsin, together with the large agricultural, biotechnological, and nutritional interest in this plant species [8,42], prompted us to select it for further structure–function analyses. Given this selection, and to be respectful to the ATSI abbreviation initially suggested by Hejgaard et al. (1994) [11] for the inhibitor from the *A. caudatus* species, we kept it throughout this work. In the next step, we conducted detailed X-ray crystallisation studies of it in complex with trypsin. This was achieved with the known model bovine trypsin, when mixed 2:1 (mol:mol) with an excess of the inhibitor, which was followed by incubation at 25 °C. The formed complex, isolated and purified by gel filtration chromatography, gave rise to well-formed crystals and excellent X-ray electron density maps, allowing us to trace the full complex structure without discontinuities. Hence, the unexpected mid-size faint bands that appeared in the electrophoretic analysis of the complex, probably due to trypsin autolytic action in the SDS-PAGE loading buffer, were not taken into consideration, as already mentioned at the end of Section 2.4. of the Results.

The excellent X-ray-derived electron density maps at 2.8 Å resolution (see Table S3 for X-ray data collection) allowed for a clean delineation of the main and side chains of both the bovine trypsin and ATSI, including the active site of the former and binding site of the latter. Remarkably, the long loop containing the binding site of the inhibitor, which extends from residues Arg40 to Arg48, appeared here intact, in spite of the fact that in other similar trypsin inhibitor/trypsin complexes [43], and in certain conditions, it becomes cleaved, from which it has been frequently referred to as the “reactive loop”. Remarkably, the crystal structure of the complex indicates that enzyme and inhibitor are in a substrate-like transition state mode, which is essential to understand the molecular mechanism of the binding, as well as for potential engineering purposes. The occurrence of the single disulfide bond of ATSI, Cys4-Cys49, in a position that constrains the binding loop by its centre is also significant, helping to define its docking into the binding sites of the enzyme. This probably contributes to its highly efficient inhibition of bovine trypsin, with a low nanomolar *K_i_* value.

The here-derived structure facilitates the classification of ATSI as a member of the structural–functional family of potato-I plant inhibitors, joining other ones from plants assigned to this family, as is the case for rBTI from Buckwheat, LUTI from *Linum usitatissimum* (var *Linum humile*), and BGIT from *Momordica charantia* (Bitter gourd), to which it aligns very well, as shown in Figure 9. The availability of the detailed three-dimensional structure of ATSI could facilitate future protein engineering studies: i.e., by adding disulfides to it in order to strongly increase its stability. Such mutations probably would improve its endurance and capability to act as an inhibitor on pest enzymes in *in vivo* transgenic plants, an application already reported for other protease inhibitors [26].

Previous evidence has indicated that ATSI from *A. caudatus* is a good enzyme inhibitor for certain mammalian serine proteases, as trypsin and chymotrypsin, as well as for *bacillus* subtilisins and a *Fusarium* proteinase, suggesting that it might act as an anti-microbial against them for the defence system of amaranth [11]. On the other hand, when growth inhibitory assays with ATSI from *A. hypochondriacus* were made on digestive proteinases in crude larvae extracts from the *P. truncatus* insect [39], which is not a usual predator of this amaranth species but has been known to have trypsin-like enzymes, positive results were observed. However, when such assays were extended to larvae extracts from other common insect grain pests, such as *Sitophilus zeamais*, *Tribolium castaneum*, *Callosobruchus maculatus*, *Zabrotes subfasciatus*, and *Acanthoscelides obtectus*, they did not show inhibition action by ATSI [39]. Also, assays from the same authors on crude fungal extracts from *Aspergillius niger* and *Aspergillius fumigatus* were negative regarding *in vitro* inhibition assays with ATSI. Later, the same laboratory found low levels of this inhibitor in young *Amaranthus hypochondriacus* sprouts, probably assignable to plant defence mechanisms but not wound-inducible ones. The study concluded that the involvement of the inhibitor in defence against bacteria and insects is still its most probable role [41]. Also, other reports have asserted the anti-microbial capability of organic and/or aqueous extracts of the shoots/sprouts, steams, leaves, and seeds from different amaranth varieties, such as *A. lividus* and *A. hybridus* [38,44] or *A. caudatus* [38,45,46], including a diversity of bacteria and fungi. Overall, evidence for the involvement of ATSI in plant defence have been controversial.

Given that the roles of ATSI in amaranth plants are still not presently well clarified, particularly regarding ATSI as an antimicrobial, as well as regarding its potential biotech-related properties, we assayed its purified form against distinct classes of microbes in culture, selecting several of them considered as models of bacterial plant pathogens: i.e., against the bacteria *Erwinia amylovora* (Ea), *Xanthomonas arboricola* pv. pruni (Xap), and *Pseudomonas syringae pv*. tomato (Pto), as well as against the plant-pathogenic fungi *Fusarium oxysporum*, *Penicillium expansumi*, and *Botrytis cinerea*. In this work, in spite of using a range of assays reaching rather high concentrations of ATSI (i.e., up to 100–200 µM), no significant inhibitory effect of ATSI on the growth of those bacterial and fungal microorganisms were detected in any of them, in contrast with the clear inhibitory effect observed when using reference model peptides (antibacterial and antifungal) that have a proven effect on such microorganisms [21,22,23]. We also observed negative results when the assays were extended to a minimal, wall-less bacteria, with poor protection against the action of harmful external molecules, such as *M. genitalium*., which was used as a model. In spite of these results, it should not be overlooked that the occurrence of diverse, important microbial pests in *Amaranthus*, among bacteria, fungi, and mycoplasmas and related phytoplasmas, has been reported [34,47], a fact that would justify the involvement of defensive molecules against it in these plants. Unfortunately, such involvement and activity has not been evidenced in the present work.

Our final assay, conducted to explore the potential anti-microbial effect of ATSI on the protozoan *Plasmodium falciparum*—the main causative agent of malaria—yielded unexpectedly negative results, despite related biotech/biomed precedents and prospects regarding protease inhibitors. Previous research had suggested that peptide or proteinaceous inhibitors of serine proteases might inhibit the growth and viability of human Plasmodium parasites [48,49,50]. However, our findings indicate that ATSI does not meet this expectation. This result is particularly notable given ATSI’s apparent haemolytic activity, which may limit its utility in therapeutic applications against this parasite. In this respect, when developing inhibitors to combat *P. falciparum*, the goal is to target the parasite without harming the host’s red blood cells. Effective inhibitors should disrupt the parasite’s lifecycle or its ability to infect and multiply within red blood cells. However, if an inhibitor also damages red blood cells or causes haemolysis (the rupture of red blood cells), it could exacerbate the condition, leading to severe anaemia and other complications.

Certainly, the results here collected on the effects of ATSI on distinct microbial cell cultures cannot be generalised or considered as firm proof of its lack of a role as an antimicrobial in amaranth cultivars, given that the assays were not made against the specific microbes that actually infect amaranth plants in the fields, whether bacteria, fungi, or phytoplasma [34,51,52]. The present lack of well-established cell cultures for the microbes naturally infecting amaranth makes such assays quite difficult, but performing assays on these microbes will be worthwhile when feasible. However, overall, the here-collected evidence seems to disfavour the role of ATSI, and its potential use, as an antimicrobial and suggests that functional and applicative research in this area should be redirected towards defensive insect deterrence roles and/or other protective/regulatory functions in amaranth seeds.

## 4. Materials and Methods

### 4.1. Materials

*Seeds:* A set of four certified Andean plant seeds, of *Chenopodium* spp. (quinoa), *Amaranthus hybridus* (sangorache), *Amarantus caudatus* (kiwicha), and *Lupinus mutabilis* (lupino), were supplied by the INIAP, Instituto Nacional de Investigaciones Agropecuarias de Ecuador.

*Substrates:* Nα-Benzoyl-l-arginine-4-nitroanilide-hydrochloride (bApNA), Suc-Ala- Ala-Pro-Phe 4-nitroanilide (sAAPFpNA), and Benzylocarbonyl-glycyl-glycyl-L-Leucine 4-nitroanilide (bGGLpNA) were obtained from Bachem (Bubendorf, Switzerland).

*Target proteases:* Bovine trypsin, α-chymotrypsin, and subtilisin A from Bacillus licheniformis were provided by Sigma-Aldrich (Saint Louis, MO, USA).

*Bovine trypsin immobilization reagents:* Sepharose^®^ CL4B, glycidol, sodium borohydride, and sodium periodate were purchased from Sigma-Aldrich (Saint Louis, MO, USA).

*Bovine trypsin immobilization on glyoxal Sepharose^®^ support:* The immobilization process was carried out on bovine trypsin as described in [53], including the preparation in our lab of the glyoxal derivative and of an enzyme immobilization stage by multipoint covalent attachment to the support [54].

### 4.2. Methods

#### 4.2.1. Extraction from Seeds

*Extract preparation*: Seeds (50 g per experiment) were ground into a fine flour (particle size ≤ 1 mm), and pre-degreased with 1-propanol (Promega, Madison, WI, USA) at a 1:4 ratio (solid–liquid) [55]. Extracts were kept at 4 °C until they were assayed for trypsin inhibitory activity by resuspending 1 g (dry weight) in 5 mL of 50 mM Tris-HCl buffer, pH = 8.0, 150 mM NaCl, which was followed by centrifugation at 13,000 rpm for 15 min at 4 °C, recovery of the supernatant fraction (crude plant extract), and storage at −20 °C.

*Extract treatments*: For the inhibitor preparation studies (but not analysis), crude plant extracts were submitted to two different cleaning treatments: heat treatment (65 °C, 30 min) and acid treatment (TCA 2.5% *w*/*v*, 4 °C, 30 min). At the end of the acid treatment, the sample was neutralized with 2 M Tris-HCl buffer, pH = 8.0. In both treatments, the final sample was centrifuged at 13,000 rpm for 15 min at 4 °C, and the supernatant was recovered and stored at −20 °C for further analyses.

*Protein concentration and SDS-PAGE analyses*: Total protein concentration was measured in the crude and treated extracts using the bicinchoninic acid (BCA) method [56]. Polyacrylamide gel electrophoresis analyses were carried out on pre-cast or home-made 15% acrylamide gels, as described in [57]. The gels were stained with Coomassie Blue R-250.

#### 4.2.2. Enzymatic Characterization of Proteolytic and Inhibitory Activities

*Trypsin inhibition assays:* Trypsin inhibitory activity was assayed for all extracts, subject to the experimental condition that enzyme (E), substrate (S) and inhibitor (I) concentrations as well as the [I]/[E] ratio and preincubation time followed the requirements for tight-binding inhibitor assays [58]. All assays were performed in triplicate at 37 °C in 96-well plates using a multiplate reader Wallac 1420 VICTOR3 (PerkinElmer, Waltham, MA, USA), in a final reaction volume of 250 μL. The reactions were followed at 60 s intervals for 1 h and recorded as initial velocities. In the protease inhibition assays, mixtures of activity buffer, biological sample, and enzyme were preincubated at 37 °C for 15 min before substrate addition. Other experimental conditions are listed as follows: 0.1 µM trypsin; 1.0 mM bApNA substrate (at 1K_m_) and 20 mM Tris-HCl buffer; pH = 8.0; 150 mM NaCl, 20 mM CaCl_2_, and 0.05% *v*/*v* Triton X-100 as activity buffer [59]. Related abbreviations: Res.E.A.: residual enzymatic activity; V_i_/V_0_: fraction of enzymatic activity in the presence (V_i_) and absence of inhibitor (V_0_) in term of initial velocities; IA: inhibitory activity; and SIA: specific inhibitory activity. One unit of inhibitory activity (IA, equivalent to V_i_) was defined as the amount of inhibitor able to reduce one unit of enzymatic activity (equivalent to V_0_), (V_i_/V_0_) representing the residual enzymatic activity. Specific inhibitory activities (SIAs) were derived by dividing the inhibitory activity by the protein concentration, both from the same sample.

*α-Chymotrypsin and Subtilisin inhibition assays*: For α-chymotrypsin from bovine pancreas, assays were performed at 0.2 µM, in 50 mM Tris-HCl, at pH 8.0, using 1.0 mM sAAPFpNA as a substrate (at 1K_m_) [60]. For subtilisin A, assays were performed at 0.4 µM in 50 mM Tris-HCl, at pH 8.5, containing 10% *v*/*v* DMSO, using 0.4 mM as a substrate (at 0.5K_m_) [61].

*Dose–response and IC_50_ values:* Dose–response relationships were obtained by measuring the inhibitory activity at different extract concentrations. The IC_50_ value was calculated using the GraphPad Prism 5 software (GraphPad Software Inc., San Diego, CA, USA) at *p* < 0.05.

*Determination of K_i_ of natural ATSI against bovine trypsin:* The *K_i_* value of natural ATSI against bovine trypsin was determined by measuring the enzymatic residual activity (V_i_/V_0_) at different inhibitor concentrations and using a fixed enzyme and substrate concentrations, as described above, in terms of initial velocities. bApNA was used as a substrate for these analyses, in 20 mM Tris-HCl buffer, where pH = 8.0, with 150 mM NaCl, 20 mM CaCl_2_, and 0.05% *v*/*v* Triton X-100 as the activity buffer [59], at 37 °C. The determination of *K_i_* values was carried out using a determined preincubation time of 15 min, required to establish an enzyme–inhibitor equilibrium. The actual and best estimate of *K_i_* value was obtained by fitting the experimental data to the equation for tight-binding inhibitors described by Morrison and Copeland [14,15], by non-linear regression using the GraphPad Prisma 5 software (GraphPad Software, Inc., San Diego, CA, USA) at *p* < 0.05. Data were means and (n = 3) S.D.

#### 4.2.3. Molecular Characterization of Protease Inhibitors

*MALDI.MS matrices and MS sample treatments:* Matrix-assisted laser desorption ionization (MALDI), in time-of-flight mode (TOF) for mass spectrometry (MS), was performed for the analysis of seeds extracts, proteins, and protein trypsin inhibitors. 2,6-dihydroxy-acetophenone (DHAP) and 2,5-dihydroxy-benzoic acid (DHB) matrices for sample preparation were provided by Sigma-Aldrich (Saint Louis, MO, USA). Unless otherwise indicated, 1 μL of sample was deposited on a MTP 384 target plate ground steel TF (Bruker Daltonics, Bremen, Germany), followed by the deposition of 1 μL of DHAP/DHB as a matrix. The mixture was allowed to dry at room temperature.

*Spectra acquisition for MALDI-TOF MS and IF-MALDI.MS experiments:* MALDI-TOF.MS mass spectra were manually acquired, with the following parameters: initial and maximal power laser of 60 and 100%, respectively; range from 1000 to 20,000 *m*/*z* in linear mode geometry; extraction delay time set at 250 ns; acceleration voltage operating in positive ion mode of 25 kV; 0.5 Gs/s sampling rate digitizer; time ion selector deflection of mass ions 1000 *m*/*z*. To improve the signal-to-noise ratio, 3000 laser shot steps were acquired for each mass spectrum. An external calibration was performed using a standard protein calibration mixture (Protein Calibration Standard I, Bruker Daltonics, Bremen Germany). All data were reprocessed using the Flex Analysis software version 3.3 and updated (Bruker Daltonics, Bremen, Germany).

*Identification of trypsin inhibitors by intensity-fading MALDI-TOF MS in crude plant extracts:* Two independent biological replicates of all the selected extracts were subjected to IF-MALDI.MS analyses using our reported indirect detection method based on an affinity step [12,62] with immobilized bovine trypsin on glyoxal-Sepharose^®^ CL4B, followed by MALDI-TOF MS analysis, with or without previous C18 RP-HPLC analysis, according to need. After optimizing the MALDI spectrum of the biological sample (total spectra), experimental conditions were set in order to achieve the initial retention of inhibitor molecules on the affinity matrix, followed by five washing steps using the interaction buffer to remove sample excess or components bound by weak or non-specific interaction. The process was performed either in home-made microcolumns, of 200 µL each, or on micro-centrifugal spin cells or microbeads [13]. A final elution step with 0.5% TFA was performed for 10 min to recover the strong ligand(s). Each step was analysed by MALDI-TOF MS using three replicates of each fraction, previously desalted using ZipTip^®^C_4_ pipette tips. Other experimental details are described in [13].

*Purification of natural ATSI:* After the preparation of crude extracts of *A. caudatus* seeds (described above), the sample was solubilized in the column equilibration buffer (20 mM Tris-HCl, pH = 8.0, 150 mM NaCl, 20 mM CaCl_2_), and the solution was centrifuged again and loaded on a bovine trypsin-glyoxal Sepharose^®^ CL4B column (1.6 cm × 10 cm, containing 1.8 mg of trypsin/mL gel), previously equilibrated at 1 mL/min with equilibration buffer. Non-retained molecules were removed by washing the column with 10 volumes (250 mL) of the same equilibration buffer. Elution was then performed with 10 volumes of 50 mM glycine-HCl buffer, pH = 2.0, at 1 mL/min. The whole process was carried out at room temperature. The chromatogram was conducted at 280 nm in an ÄKTA Purifier FPLC system (GE, Chicago, IL, USA), and the fractions corresponding to the elution peak were collected in tubes containing a neutralizing buffer. The purity of ATSI was determined by RP-HPLC on a C8 column, by MALDI-TOF MS to derive its molecular mass, and by Tris-glycine-SDS-PAGE.

##### Determination of the Number of Free and Total Cysteine Residues

*Free cysteine residues:* Purified native ATSI was dissolved in 6 M guanidine chloride and 100 mM ammonium bicarbonate, pH = 8.0, and treated with iodoacetamide to a final concentration of 50 mM, at room temperature, for 30 min, in the dark.

*Total cysteine residues:* Purified native ATSI was dissolved in 6 M guanidine chloride and 100 mM ammonium bicarbonate, pH = 8.0, and heated at 95 °C for 5 min. Then, the sample was equilibrated at 60 °C, adjusted to 20 mM in DTT, and reduced at the same temperature for 30 min. Subsequently, it was brought to room temperature and S-carbamidomethylated with 50 mM iodoacetamide for 30 min, in the dark. At the end, both samples were acidified with TFA, desalted by analytical RP-HPLC using a Jupiter C_4_ column (Phenomenex, Torrance, CA, USA), and analysed by MALDI-TOF MS.

##### MALDI Top-Down Sequencing Using ISD for Natural ATS

*ISD fragmentation:* The purified, reduced, and S-carbamidomethylated ATSI was fragmented using a ISD (in-source decay fragmentation) reflector approach [10,63]. Spectra were manually acquired using the AutoExecute™ acquisition control program, with the following parameters: initial and maximal power laser of 40 and 100%, respectively; range from 1000 to 8000 *m*/*z* in reflectron mode; extraction delay time set at 100 ns; accelerating voltage operating in positive ion mode at 25 kV; 4 Gs/s sampling rate digitizer; time ion selector deflection of mass ions at 1000 *m*/*z*. To improve the signal-to-noise ratio, 6000 laser shots were acquired for each mass spectrum. An external calibration was applied using a standard peptide calibration mixture (Peptide Calibration Standard, Bruker Daltonics, Bremen, Germany). MALDI.MS analyses were performed in an UltraflexExtreme MALDI-TOF/TOF spectrometer (Bruker Daltonics, Bremen, Germany).

*Sequencing analysis:* Monoisotopic masses were determined using the Flex Analysis 3.3 software (Bruker Daltonics, Bremen, Germany) with the SNAP peak-picking algorithm. The fragmentation spectrum was analysed using the Biotools software (version 3.2, Bruker Daltonics). The ISD spectrum was submitted to the Mascot search engine. In all searches, carbamidomethyl (C) was established as a global modification. For y-ion spectra, any variable modification was set, while searches based on c-ions required selecting “Amide (C-Term)” as the variable modification. In the case of identification based on a- or z+ 2-ions, the “ISD a-series (C-Term)” or the “ISD z+2-series (N-Term)” was selected.

#### 4.2.4. Preparation of ATSI-Trypsin Complex, Crystallization, and Structure Determination

*Formation and purification of ATSI-bovine trypsin complex:* The formation and purification of the bTrypsin-ATSI complex were performed by preincubation of both proteins for 30 min in 20 mM Tris-HCl buffer, pH = 8.0, with 150 mM NaCl and 20 mM CaCl_2_, all at room temperature. For this purpose, 13.3 mg of bTrypsin was incubated with 9.0 mg of pure natural ATSI for a final reaction volume of 10 mL (equivalent to an enzyme/inhibitor molar ratio of 1:2). The complex was captured on a size-exclusion chromatography column (HiLoad Superdex 75, 2.6 × 60 cm, GE Healthcare) equilibrated with the same buffer used for complex formation and run at 1 mL/min. Elution peaks corresponding to the complex and inhibitor were analysed by SDS-PAGE at 15% acrylamide. The bTrypsin-ATSI complex was concentrated to 16.0 mg/mL using an Amicon Ultra-4 centrifugal filter MWCO 3 kDa (Millipore, Darmstadt, Germany) and, at this time, the remnant size exclusion chromatography buffer was changed to 5 mM Tris-HCl, pH = 8.0, containing 50 mM NaCl.

*Crystallization and Data Collection*: Crystals of the complex between ATSI and bTrypsin were obtained at 18 °C using hanging-drop vapour diffusion methods in a buffer containing 2 M AmSO_4_, 0.1 M HEPES pH 7.5, 2% PEG400. Single crystals appeared after a few days. Crystals were cryoprotected in reservoir buffer containing 15% glycerol or ethylene glycol flash-cooled in liquid nitrogen prior to diffraction analysis. Diffraction data were recorded from cryo-cooled crystals (100 K) at ALBA beamline XALOC-BL13 [64]. Data were integrated and merged using XDS [65] and scaled, reduced, and further analysed using CCP4 [66].

*Structure Determination and Refinement:* The structure of the complex ATSI-bovine trypsin was determined from the X-ray data at 2.85 Å resolution by molecular replacement using the PDB from bovine trypsin (PDB code 1AQ7) as a model. Manual building and improvement of the model was performed using the program COOT [67]. Refinement was performed using Phenix [68]. Ramachandran analysis showed that 91.43% of the residues (2092) were in the preferred regions, 7.65% of residues (175) were in allowed regions, and 0.92% of residues (21) were in outlier regions, for all complexes of ATSI-trypsin in the asymmetric unit. For data collection, quality of data analysis, and Xray crystallographic statistics, refer to Table S3. All structural figures were prepared using PyMOL 3.1 vs. software (http://www.pymol.org, accessed last time on 22 December 2024).

#### 4.2.5. Cell Culture and in Cellulo Inhibitory Assays

*Bacterial and fungal strains and growth conditions:* The following bacterial and fungal pathogens were used: *Penicillium expansum* EPS 26 (INTEA, University of Girona), *Fusarium oxysporum* f. sp. *lycopersici* FOL 3 race 2 (ATCC 201829; American Type Culture Collection), *Botrytis cinerea* (CECT 20518; Spanish Type Culture Collection), *Erwinia amylovora* EPS 101 (INTEA, University of Girona), *Xanthomonas arboricola* pv. pruni (CFBP 5563, French collection of plant pathogenic bacteria), *Pseudomonas syringae* pv. tomato DC300 J. Murillo, Plant Pathology, Public University of Navarra, Spain), and *Mycoplasma genitalium* G37 strain (NCTC 10195). Non-mycoplasmas bacterial strains were cultured in LB agar for 24 h at 28 °C and scrapped from the surface to obtain suspensions adjusted to 5.10^7^ CFU/mL. Fungal strains were grown on PDA agar for 7–10 days at 23 °C. Spores/conidia were collected by spreading sterile distilled water onto the surface of the plate. The spore suspension was filtered through three layers of sterile cheesecloth and adjusted to 1 × 10^4^ spores/mL. *M. genitalium* was grown for 3–4 days in SP4 medium using tissue culture flasks incubated at 37 °C and 5% CO_2_ [69]. The culture buffer used was PBS, at pH 7.4 for non-mycoplasma bacteria and fungi, and at pH 7.8 for mycoplasma; in the former two cases, alternative experiments run in citrate–succinate–phosphate buffer, at pH 7.0, gave rise similar to results to PBS.

*Antimicrobial activity of ATSI:* For growth inhibition assays for ATSI, agar spot tests in LB in the case of bacterial growth (non-mycoplasma) were used, while in the case of fungal growth, PDA was used. Different concentrations of ATSI were prepared in succinate–citrate–phosphate buffer at pH 7.0. To determine the antibacterial activity, LB soft agar (0.7%) was mixed with a bacterial suspension. Next, 0.5 mL of the target bacterial pathogen at 5 × 10^7^ CFU/mL was mixed in 4.5 mL of LB soft agar and overlaid on the plate containing the same media. Then, 10 µL of ATSI was deposited on the agar soft layer. For fungal inhibition assay, a fungal suspension at 10^4^ spores/mL was spread on a PDA plate, and 10 µL of ATSI was added to the agar surface. Three replicates, as well as positive and negative controls for the products, concentrations, and buffers, were included for each assay. The plates were incubated at 28 °C for 24–48 h (bacterial growth) or at 23 °C for 4–7 days (fungal growth), and microbial growth was determined qualitatively through the presence of inhibition zones.

Regarding the tests on *P. falciparum*, its culture conditions in red cells, the details of the assays on the potential inhibitory effects of ATSI, and comparative studies with the reference antimalarial compound chloroquine have been previously described in [70].

To test the inhibition of *Mycoplasma genitalium* growth, 190 µL of 1/3 serial dilutions of a stock at 1 × 10^8^ CFU/mL in SP4 medium were placed in the wells of a 96-well TPP tissue culture plate containing 10 µL of several inhibitor dilutions in PBS. The plate was placed into a Sunrise Absorbance Microplate Reader (Tecan) and incubated at 37 °C for 8 days, with *A*_550_ absorbance readings taken every 15 min. Absorbance readings measured the culture acidification resulting from mycoplasma growth, which turns the colour of the phenol indicator from red violet to orange yellow [71]. The inhibition values were computed using the following equation: //Inhibition = 1 − (*Ais*-*Afs*)/(*Aic*-*Afc*)//, where *Ais* is the absorbance of the starting culture in the presence of the inhibitor; Afs is the absorbance at the latter stages of the culture in the presence of the inhibitor; *Aic* is the absorbance of the starting culture in the absence of the inhibitor; and *Afc* is the absorbance at the latter stages of the culture in the absence of the inhibitor. All inhibition assays were performed by using four biological replicates.

## 5. Conclusions

A comparative analysis of seeds from *Lupinus mutabilis* (lupine), *Chenopodium quinoa* (quinoa), and two amaranth species, *Amaranthus hybridus* and *Amaranthus caudatus*, revealed the presence of protein trypsin inhibitors in them with molecular masses of 4–8 kDa. Through defatting, affinity chromatography and identification by HPLC and IF mass spectrometry, the two amaranth species showed the highest inhibitory content, with an identical inhibitor of sixty-nine residues, with one disulfide, a molecular mass of 7889.1 Da, and matching mass fragmentation profiles. In contrast, the lupine and quinoa inhibitors were heterogeneous. The *Amaranthus caudatus* inhibitor in complex with bovine trypsin was structurally characterized by X-ray crystallography. It exhibited a potato-I-type/clan fold, with its conformation trapped in a substrate-like transition state mode. Its reactive loop (Arg40-Arg48) was constrained by a disulfide bond, likely contributing to its high trypsin affinity (*K_i_* at low nanomolar level). Cell growth assays indicated a lack of efficacy against diverse plant microbial pathogens, suggesting the redirection of future research on its potential roles towards insect deterrence or other defensive/regulatory roles.

## Figures and Tables

**Table 1:** Summary of the purification procedure of ATSI from *A. caudatus* [data are means (n = 3) ± S.D.].

	Total Protein (mg)	Inhibitory Activity (U)	Specific Inhibitory Activity (U/mg)	Yield (%)	Purification (Fold)
Crude extract	186.5	11.1 ± 1.5	0.059 ± 0.008	100	1.0
Peak from affinity chromatography	1.88	8.5 ± 0.7	4.5 ± 0.3	76.4	75.7

## Data Availability

The important data of this work are contained within the article and Supplementary Materials.

## Supplementary Materials

The following supporting information can be downloaded at: https://www.mdpi.com/article/10.3390/ijms26031150/s1. Reference [72] is cited in the Supplementary Material.
