# Supplementary material for: Analysis of Protein Inhibitors of Trypsin in Quinoa, Amaranth and Lupine Seeds. Selection and Deep Structure–Function Characterization of the Amaranthus caudatus Species
Source: Int J Mol Sci. 2025 Jan 28;26(3):1150. doi: 10.3390/ijms26031150 (PMC11817793; doi:10.3390/ijms26031150)
Supplement: Supplementary file 1 [file ijms-26-01150-s001.zip › ijms-3390330-supplementary.pdf]

SUPPLEMENTARY MATERIALS (Figures and Tables)

Table S1. Protein content in the crude and treated seed extracts

	**Protein concentration (mg/mL)**			
**Sample**	**Quinoa**	**Lupino**	***A. hybridus***	***A. Caudatus***
Crude extract	5.1 ± 0.4	24.3 ± 2.1	3.7 ± 0.3	4.7 ± 0.3
Heat-treated extract	5.1 ± 0.4	23.9 ± 1.9	3.6 ± 0.2	4.9 ± 0.4
TCA-treated extract	4.9 ± 0.1	18.8 ± 0.5	3.31 ± 0.02	4.5 ± 0.1

Results derived from BCA-based analyses. Data are means (n=3) ± S.D.

Table S2. Immobilization parameters of bovine trypsin on glyoxal-Sepharose® CL-4B

**$Prot_{initial}$ (mg)**	**$Prot_{final}$ (mg)**	**$Prot_{immobilised}$ (mg)**	**V support (mL)**	**DI prot (mg prot/mL gel)**	**%I prot**
88.9	15.0	73.9	40	1.8	83.1

Prot: protein. V=volume. %I prot: percentage of enzyme immobilization in terms of protein. DI prot: degree of immobilization in terms of protein. Data are means (n=3). Immobilization of bovine trypsin on glyoxal Sepharose® CL-4B support was carried out at 4ºC for 3h. At the end of immobilization time, %I prot of 83.1 was achieved, representing a DI prot of 1.8 mg bovine trypsin / mL gel.

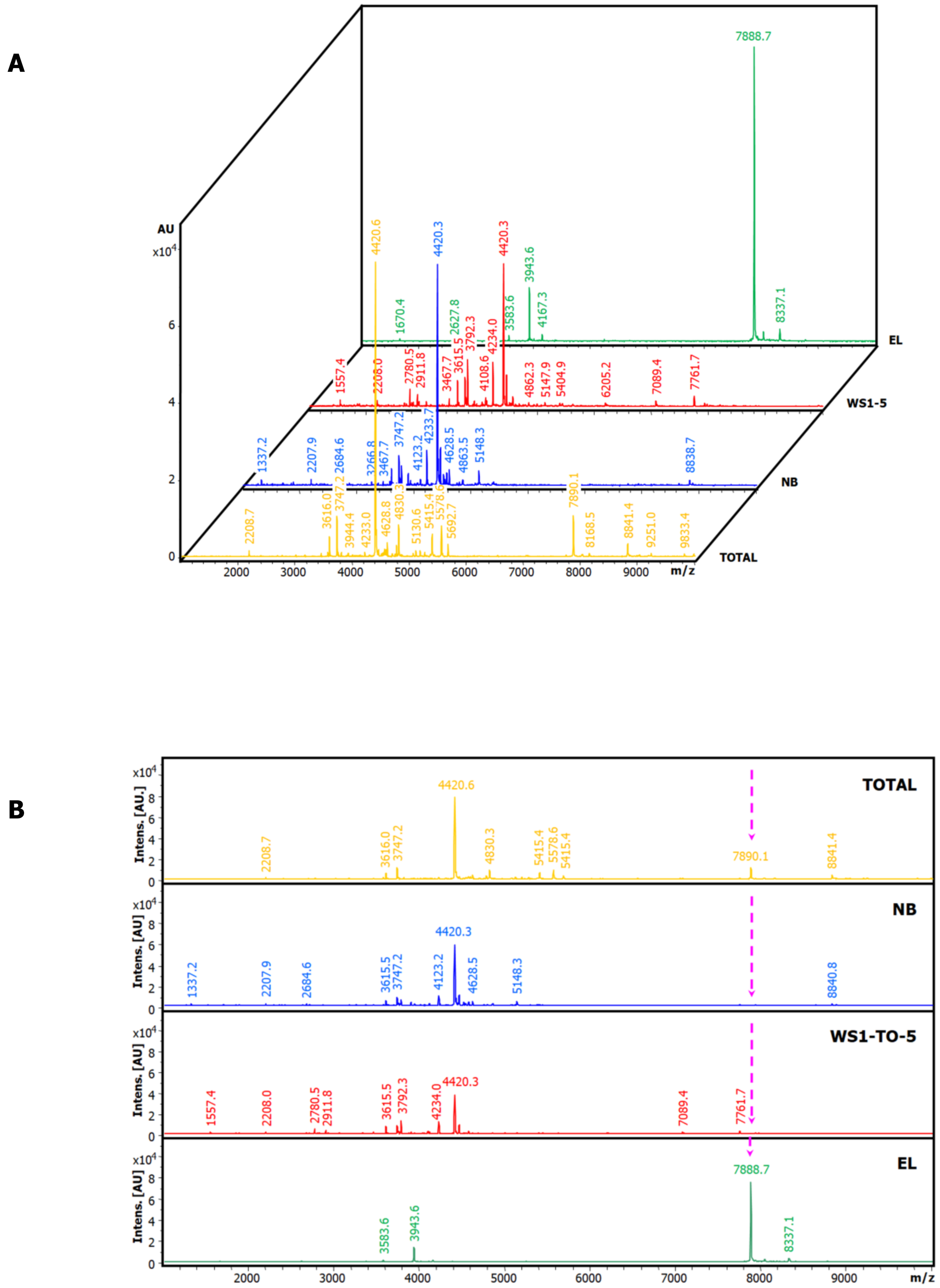

Figure S1. Intensity-Fading MALDI-TOF MS of *A. caudatus* crude extract on bovine trypsin-glyoxal Sepharose® CL-4B. Assay with trypsin in the matrix. (A) Stack and (B) detailed view of the mass spectra. TOTAL: crude extract of *A. caudatus* in 20 mM Tris-HCl buffer pH=8.0, containing 150 mM NaCl and 20 mM $CaCl_2$. NB: non-binding molecules interaction at pH=7.5, 10 min. WS1-TO-5: washing at pH=8.0. EL: elution with 0.5% v/v TFA, 10 min. Interaction assays were carried out in duplicate at room temperature. It should be noted that the species at 7888-7990 m/z range display strong signal at the eluate fraction and weak with the initial extract (Total), or null at washings, indicative that the derivatisation of the matrix with trypsin generates a proper affinity support.

A

B

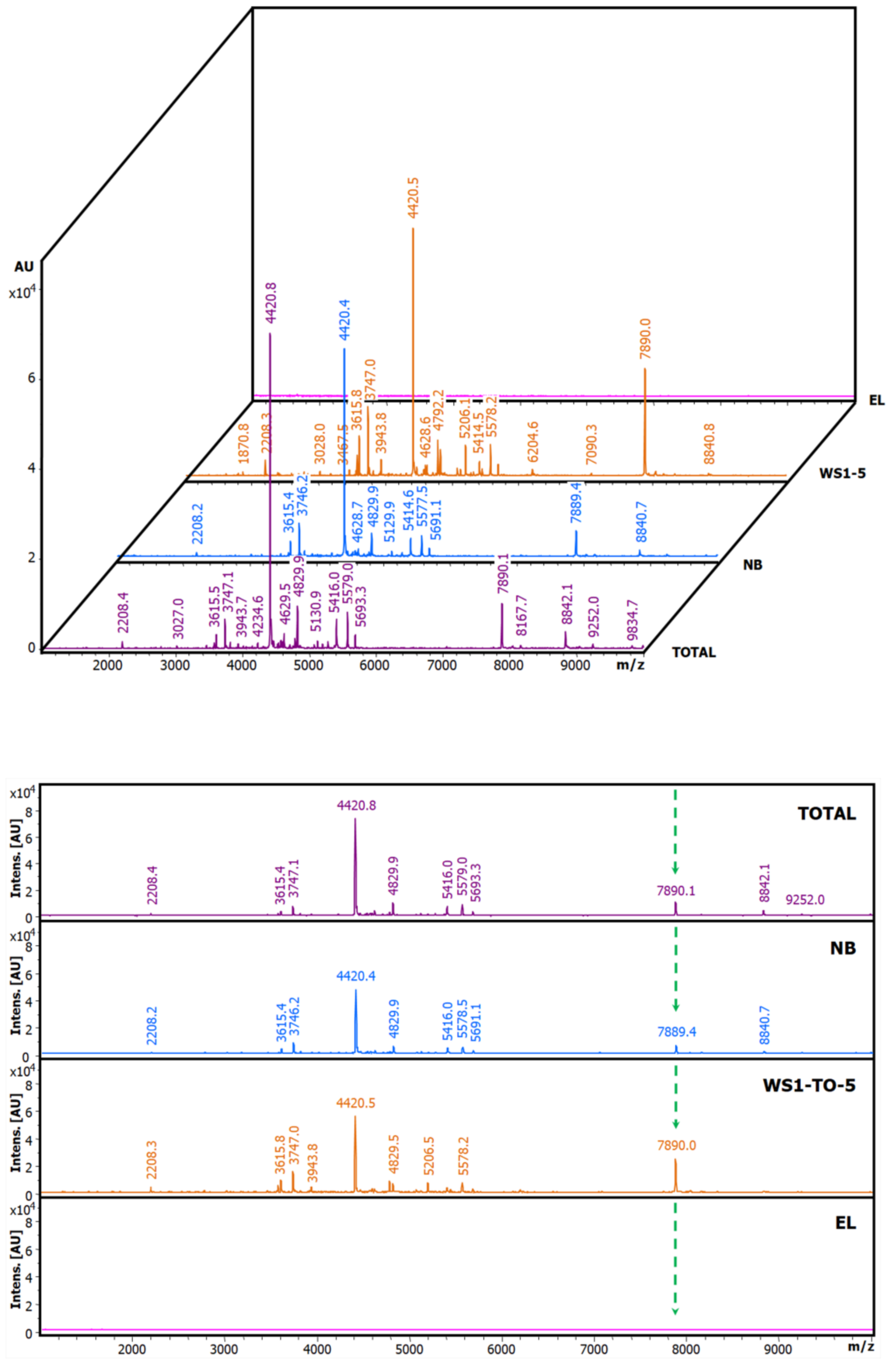

Figure S2. Intensity-Fading MALDI-TOF MS of *A. caudatus* crude extract on Sepharose® CL-4B. Assay with matrix without trypsin. (A) Stack and (B) detailed view of the mass spectra. TOTAL: crude extract of *A. caudatus* in 20 mM Tris-HCl buffer pH=8.0, containing 150 mM NaCl and 20 mM $CaCl_2$. NB: non-binding molecules, interaction at pH=7.5, 10 min. WS1-TO-5: washing at pH=8.0. EL: elution with 0.5% v/v TFA, 10 min. Interaction assays were carried out in duplicate at room temperature. It should be noted that the species at 7888-7990 m/z range don't appear in the eluate and are very faint in the initial extract, non-bound fraction and washes. Therefore, the matrix by itself has very little capability to capture ATSI.

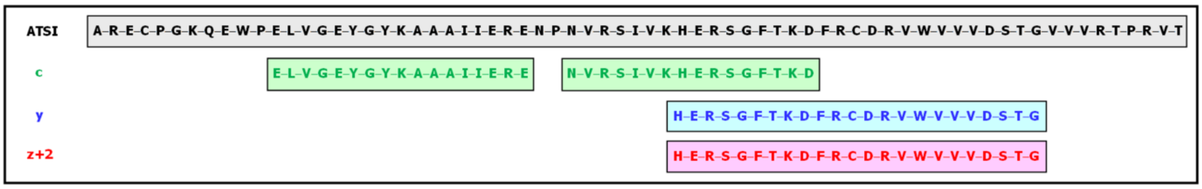

Figure S3. ISD MALDI-TOF MS analysis of ATSI. Sequence coverage of ATSI using ISD approach. Spectra were collected for the reduced and S-carbamidomethylated ATSI using 2,5-DHB as a MALDI matrix.

Table S3. Xray crystallographic statistics of the ATSI-trypsin complex.

**Data collection**	
Space group	P1
Unit cell parameters (Å)	57.29, 92.59, 108.54
Unit cell parameters (º)	90.04, 89.86, 90.03
Wavelength (nm)	0.97918
Resolution range (Å)	46.84- 2.84
R_{merge}	0.11 (0.47)
R_{pim}	0.10’ (0.42)
(I/σ(I))	4.4 (1.5)
Completeness (%)	96.6 (90.6)
Multiplicity	2.0 (2.0)
CC (1/2)	0.99 (0.72)
**Structure refinement**	
Resolution range (Å)	46.84-2.85
No. of unique reflections	50455
R_{work} / R_{free} (%)	27.4 / 33.1
No. of atoms	
Protein	17481
Overall B factors (Å^2)	46.0
Rms deviations	
Bonds (Å)	0.005
Angles (°)	1.081
**PDB code**	7AL8

(values in parentheses refer to the outer shell)

Figure S4. Effect of ATSI on the growth of a series of bacterial cell cultures from well known plant pathogens, as *Erwinia amylovora* (Ea), *Xanthomonas arboricola* pv. pruni (Xap) and *Pseudomonas syringae* pv . tomato (Pto). Different concentrations of ATSI were assayed (1-100 microM, the latter shown here), in parallel to well known reference anti-microbials. AMP, antimicrobial peptide, as a control; CNT or NTC, non-treated control. Experimental details in [21-22].

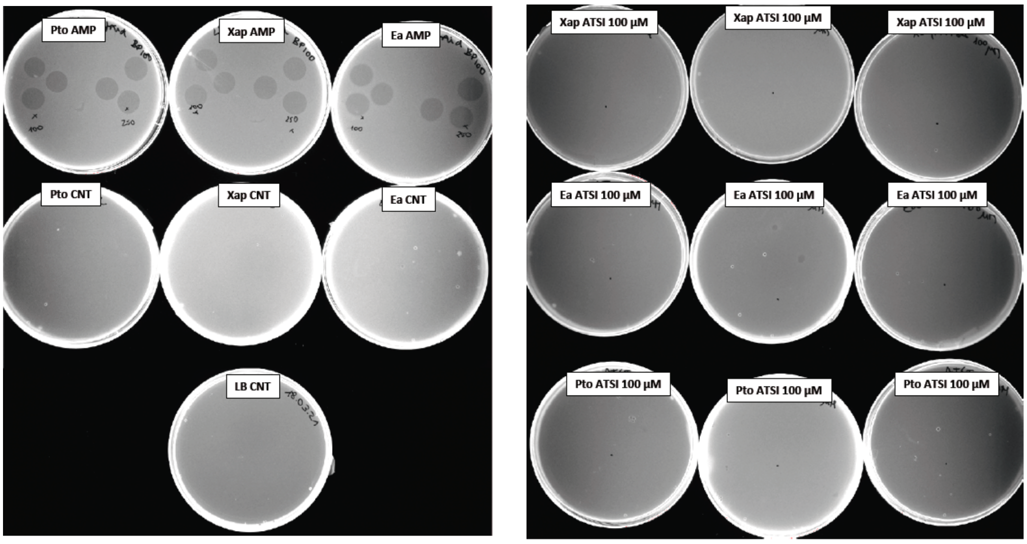

Figure S5. **Effect of ATSI on the growth of a series of cell cultures from well known fungi vegetal pests**. Assays on cultures of *Fusarium oxysporum (Fox), Penicillium expansum (Pe)* and *Botrytis cinerea (Bc)*. Different concentrations of ATSI were assayed (1-100 microM, the latter shown here), in parallel to well known reference fungi anti-microbials. AMP, antimicrobial peptide, as a control; CNT or NTC, non-treated control. Experimental details in [21-24].

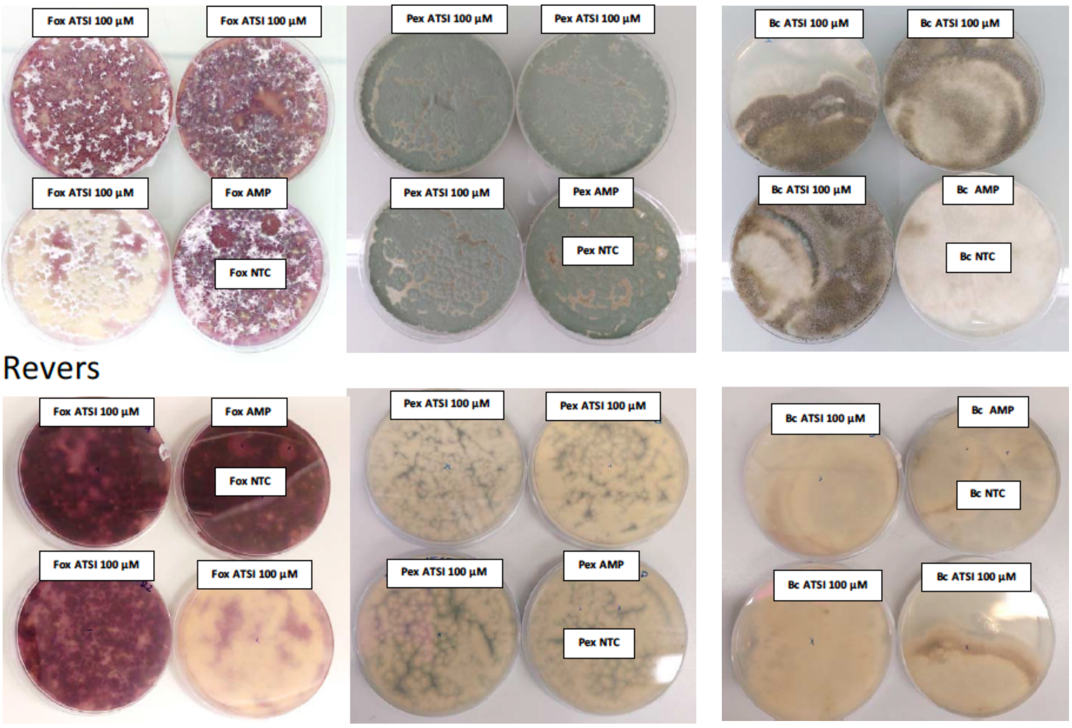

Figure S6. Effect of ATSI on the growth of *Mycoplasma genitalium* G37 cells. A) Growth curves obtained by plotting the A_{550} intensities of mycoplasma cell cultures when adding 1/20 volume of PBS (negative control, in green), in the presence of 100 µM ATSI in PBS (in blue) and adding 1/20 volume of a protein extract from *Nerita peloronta* in PBS (positive control, in red). Note that the vertical axis is in decreasing absorbance units since the colour of the culture medium turns from red-violet to orange-yellow upon mycoplasma growth. B) Inhibition of mycoplasma growth computed from the starting and ending absorbance intensities in A.

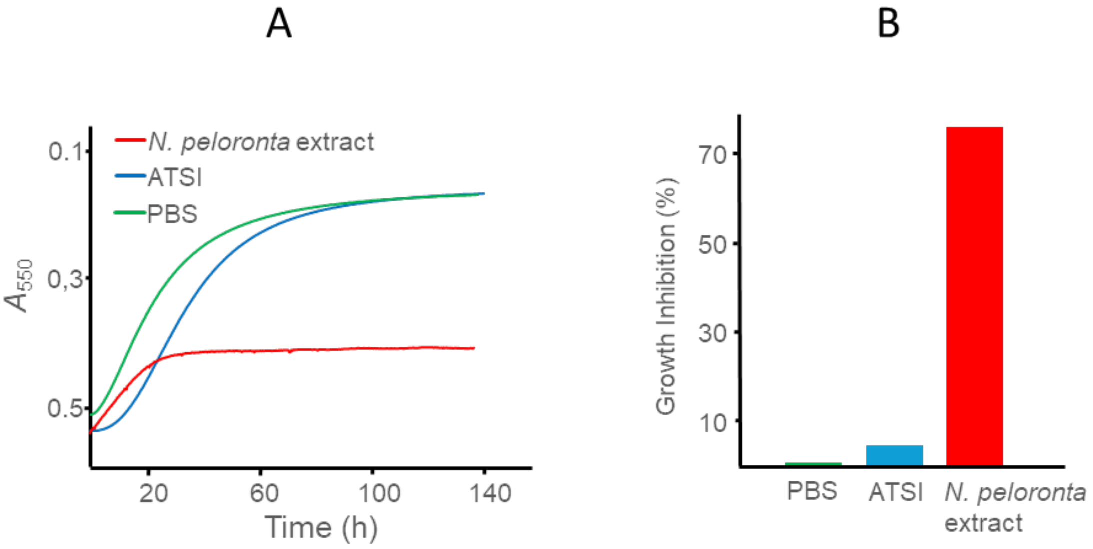

Table S4. Comparative quantitative analysis of ATSI and chloroquine on *P. falciparum* Dd2 parasitemia inhibition on human erythrocytes. The lack of effect of the ATSI trypsin inhibitor on *P. falciparum* Dd2 parasitemia growth is evidenced along a wide range of trypsin inhibitor concentrations. At the highest concentration assayed, 444 µM in ATSI, the analysis was obscured, probably due to erythrocyte haemolysis, as shown in Fig.S8.

Compound	Effector level (µM)	Parasitemia (%)
Chloroquine	100	0
	10	0
	1	2.1
	0.1	4.5
	0.01	4.1
ATSI	444	haemolysis
	222	3.9
	101	4.5
	50.5	3.8
	13	4.2
	7.5	4.7
	0	4.1
Control	0	0

Figure S7. Comparative visual effect of ATSI on *P. falciparum* Dd2 parasitemia on human erythrocytes. The visual appearance of supernatants from the control (a), and ATSI-treated red cell samples (b) is shown. The latter displayed a reddish supernatant, indicative of haemolysis of the erythrocytes at the highest concentration of trypsin inhibitor used (444 µM).

(a) (b)

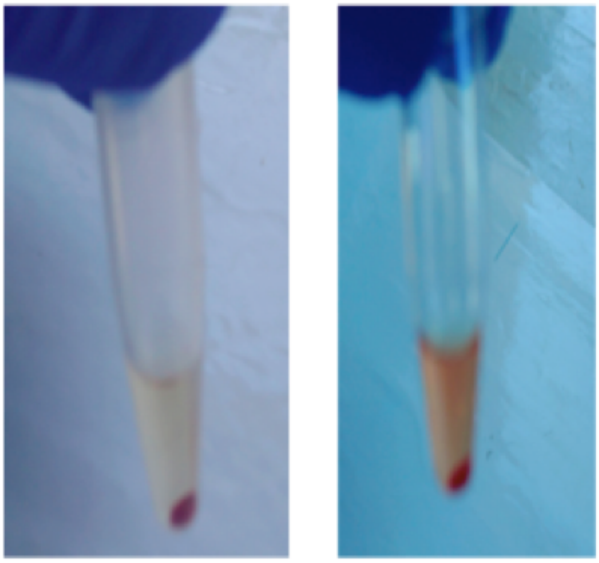

Figure S8. Alignments of the various protein reported sequences of the amaranth trypsin inhibitor, including information on potential isoforms, as well as a related plant trypsin inhibitor used as reference. (1) *Cucurbita maxima* trypsin inhibitor V [72]; 2) cDNA encoded sequence of *Amaranthus hypochondriacus* trypsin inhibitor, from [41]; 3) *Amaranthus caudatus* trypsin inhibitor, from [11]; 4) *Amaranthus caudatus* trypsin inhibitor, from the present work. The residues in blue refer to putative homologous variants; in red, two duplications are highlighted at positions 41 and 65 of the trypsin inhibitor of *Amaranthus hypochondriacus*.

[1]	SSCPGKSSW	PHLVGVGGSV	AKAIIERQNP	NVKAVILEEG	T	(40)
[2]	ARECPGKQEW	PELVGEYGYK	AAAIIERENP	NVRDIVKHER	S/Y	(41)
[3]	ARECPGKQEW	PELVGEYGYK	AAAIIERENP	NVRSIVKHER	S	(41)
[4]	ARECPGKQEW	PELVGEYGYK	AAAIIERENP	NVRSIVKHER	S	(41)

(1) contin ...	PVTKDFRCN	RVRIWVNKRG	LVVSP	PRIG	(68)
(2) ...	GFTKDFRCD	RVWVVVDYTG	VVVRT/Y	PRVT	(69)
(3) ...	GFTKDFRCD	RVWVVVDSTG	VVVRT	PRVT	(69)
(4)	GFTKDFRCD	RVWVVVDSTG	VVVRT	PRVT	(69)
